# Physical Foaming and Crosslinking of Polyethylene with Modified Talcum

**DOI:** 10.3390/polym11091472

**Published:** 2019-09-09

**Authors:** Anna Kaltenegger-Uray, Gisbert Rieß, Thomas Lucyshyn, Clemens Holzer, Wolfgang Kern

**Affiliations:** 1 Montanuniversität Leoben, Department of Polymer Engineering and Science, Chair of Chemistry of Polymeric Materials, 8700 Leoben, Austria; 2 Montanuniversität Leoben, Department of Polymeric Engineering and Science, Chair of Polymer Processing, 8700 Leoben, Austria

**Keywords:** crosslinking, foaming, nucleating agent, polyethylene

## Abstract

The aim of this study was the investigation of the use of modified talcum for supporting crosslinking and as novel nucleating agent for physical foaming of polyethylene. For the modification of the talcum, a thermal initiator was linked to the talcum surface. During the extrusion process, the initiator decomposes, and gas and radicals are formed. The gas generates the nucleation of cells and the radicals support the crosslinking process between the polymer chains. The modification of the talcum was performed in three steps: The first step was the grafting of alkoxysilanes onto the talcum surface. The second step was the chlorination of the thermal initiator for an easier linkage, and the last step was the linking between the initiator and the silanes grafted onto the talcum surface. For this study, two investigations were carried out. One investigation was the analysis of the crosslinking effect with the modified talcum. For this purpose, polyethylene plates were compression molded and the viscoelastic properties were measured with a parallel plate rheometer. The use of the modified talcum led to a higher crosslinking density. The second investigation was the physical foaming experiment in an extrusion process with nitrogen as blowing agent using both a pure and the modified talcum as nucleating agents. The foamed samples were characterized in terms of density, cell size and cell density, and compared with each other. The blend with the modified nucleating agent indicated a foam structure with a smaller mean cell size and a lower density compared to the use of the pristine nucleating agent.

## 1. Introduction

Polyolefins are the most used polymers for different applications. For example, polyethylene is used in packaging industries, insulation components, medical applications, household items, and many more. However, the application of polyethylene is limited by temperature. Therefore, polyethylene is often crosslinked to be used in applications at higher temperatures [1].

Different methods are existing to crosslink polyethylene. Electron beam irradiation, for example, is a method to produce radicals without any additives [2]. A popular method is the use of peroxide. In this method, the peroxide forms radicals which eliminate hydrogen atoms to create carbon-carbon links between the polymer chain [3]. Another procedure is to combine peroxide with silane, especially alkoxysilanes. The peroxide decomposes and forms radicals, which eliminate hydrogen atoms of the polymer chains. Then the silane groups are grafted to the polymer chains and form linkages between the chains [1,3,4]. There are different types of polyethylene with different numbers and types of chain branches depending on process and catalyst [5,6]. The development of special catalysts made it possible to produce a type of polyethylene, ethylene- octene copolymer (EOC), with a molecular structure with a narrow molecular weight distribution and homogeneous distribution of the comonomer [7]. Yussuf et al. [8] compared the crosslinking of EOC and LDPE. The ethylene-octene copolymer showed a higher degree of crosslinking compared to LDPE. This is due to the different molecular structure. This new type of copolymer has a lower crystallinity than LDPE and a large number of short chain branches. A faster moisture diffusion in the solid structure and a better silane crosslinking distribution due to the lower crystallinity led to a higher degree of crosslinking. Another reason could be the higher content of tertiary carbon atoms. Tertiary carbon atoms are more likely to react with free radicals because the associated hydrogen atom is more easily abstracted than the primary and secondary carbon atoms [9,10]. 

Rising commodity prices and the demand for lighter products always lead to a steadily growing interest in foamed products. On the one hand, it is possible to save raw material and on the other hand, very special product properties are achievable. The properties of polymeric foams depend on the foam morphology, which may be influenced by the used matrix polymer, type, and amount of the used nucleating agent, as well as by the used foaming agent. Nucleating agents are very important to achieve foam morphologies with small cell sizes and high cell density [11]. Products with a high number of small closed cells are mainly used as insulating material due to their high thermal insulation capacity [12]. Typical nucleating agents are inorganic fillers such as silica, kaolin, silicates, and talcum [6]. Due to the large number of nucleating agents there are several examinations having investigated their influence on the foam structure [13,14,15]. One of the most widely used nucleating agents is talcum [11]. Due to its importance as filler there are also a lot of chemical treatments on talcum to examine the influence on its dispersion and nucleating effect [16], on its mechanical and morphological properties [17], its effect on crystallization behavior [18], and for its use in composites [19].

The purpose of this study was to modify a typical inorganic filler, talcum, which is frequently present in polyolefins as an active filler (to improve mechanical properties or as nucleating agent [6]). The modified talcum was used to further improve the crosslinking network for higher temperature stability and also as cell nucleating agent to achieve a closed foam structure with small cells and high cell density. To achieve that, a thermal initiator 4,4′-azobis(4-cyanovaleric acid) was linked to the talcum surface. This initiator was also used in some other studies for grafting of polymers on solid surfaces [20,21,22,23]. The initiator decomposes due to temperature and gas and radicals are formed. The additional gas supports the foaming process and leads to small cells and a high cell density. The additional radicals support the crosslinking process between the polymer chains. For the linkage of the initiator to the talcum surface three steps were necessary and performed according to the study of Gert Boven et al. [23]. The silane and peroxide used for the crosslinking experiments were selected on the base of good experiences [4]. Due to the good crosslinking capability of ethylene-octen copolymer, a blend of LDPE and EOC was used. In further investigations, the combination of crosslinking and foaming will be examined. For this reason, the LDPE/EOC blend was also used for the foaming tests in order to improve comparability for future investigations.

## 2. Materials and Methods 

### 2.1. Materials

In this study, a standard talcum grade by Mondo Minerals B.V. (Steinfeld, Germany) was used. For the silanization alkoxysilane (3-aminopropyl)trimethoxysilane and as solvent toluene were used. Phosphorus pentachloride (PCl_5_), dichloromethane (CH_2_Cl_2_), hexane, the thermal initiator 4,4′-azobis(4-cyanovaleric acid) (ABCA), triethylamine and ethanol were utilized in the second and third step of the modification. All chemicals were purchased from Sigma Aldrich (Vienna, Austria) and used without any purification. 

For the crosslinking and foaming experiments, a low density polyethylene (LDPE), supplied by Sabic Europe (Sittard, Netherlands), with a melt mass flow rate (MFR) of 0.65 g/10 min (at 190 °C/2.16 kg) and an ethylene octene copolymer (EOC), supplied by Dow Europe GmbH (Horgen, Switzerland), with an MFR of 0.5 g/10 min (at 190 °C/2.16 kg) were used. For crosslinking 3-Methacryloxypropyltrimethoxysilane from Wacker Silicones (Burghausen, Germany) and dicumyl peroxide from Sigma Aldrich (Vienna, Austria) were utilized.

### 2.2. Modification of Talcum

The first step in this study was the salinization of talcum. For that purpose, alkoxysilanes were grafted onto the talcum surface. At first, 100 mL of toluene and 2 g of silane were stirred for some minutes. After the addition of 5 g of talcum, the mixture was refluxed for 5 h. The salinized talcum was washed with toluene three times and dried under vacuum for 24 h at 110 °C [23].

The second step was the chlorination of the thermal initiator for an easier linkage between the functional groups of the aminosilanes and the chlorine. For that purpose, 12 g (58 mmol) of PCl_5_ dissolved in 30 mL of CH_2_Cl_2_ were added to 1.5 g (5.4 mmol) of ABCA and 15 mL of CH_2_Cl_2_ at 0 °C. The mixture was stirred overnight. The solid was filtered off and 90 mL of hexane was added. The residual dichloromethane was evaporated and 4,4′-azobis(4-cyanopentanoyl chloride) (ABCC) was filtered and dried at 0 °C under vacuum [23].

**Figure 1:**
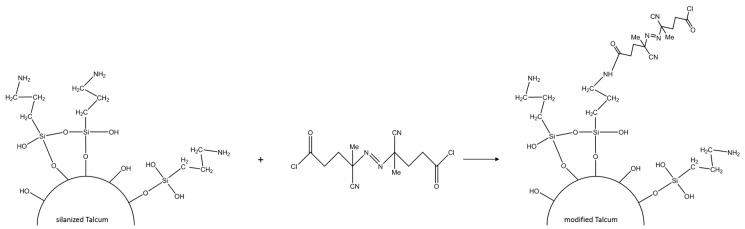
Modification of the salinized talcum surface with the initiator ABCC.

The last step was the modification of the salinized talcum (Figure 1). For that purpose, the thermal initiator was linked to the functional groups of the silane grafted onto the talcum surface. 0.75 g (2.4 mmol) of ABCC dissolved in CH_2_Cl_2_ was added dropwise to a mixture of 5 g of salinized talcum, 100 mL of CH_2_Cl_2_ and 0.4 mL of triethylamine. The mixture was stirred for approximately 3 h. The modified talcum was consecutively washed with a mixture of acidified water and ethanol, with a mixture of water and ethanol, and with ethanol [23].

For the evidence of the molecular components on the talcum particles, after each step the salinized and modified talcum was characterized with different measurement methods such as infrared spectroscopy and thermogravimetric measurement. The spectrometer Vertex 70 from Bruker (Ettlingen, Germany) was used for the IR analysis. The samples were examined without special preparation with an ATR attachment (Attenuated Total Reflection) in transmission mode. The spectra of the samples were recorded at room temperature in a spectral range of 4500–450 cm^−1^ at 16 scans with a resolution of 4 cm^−1^. Each sample was measured three times. The thermogravimetric analysis was performed with a Mettler Toledo TGA/DSC 1 (Star ^®^ System) (Mettler Toledo GmbH, Vienna, Austria) instrument. The instrument is equipped with a gas controller GC 200. The talcum samples (pure, salinized and modified talcum) were kept at 25 °C for 5 min and then heated to 600 °C with a heating rate of 10 K/min. In order to avoid oxidation reactions, the measurements were carried out in a nitrogen atmosphere with a flow rate of 20 mL/min. Three measurements were taken from each sample. 

### 2.3. Crosslinking Experiments

For the crosslinking experiments, polymer blends with a ratio of 80/20 of LDPE and EOC with 4 phr of silane (3-Methacryloxypropyltrimethoxysilane), 0.5 phr of dicumyl peroxide and with modified/pure and without talcum were produced in a kneader (Haake Rheocord 3000/600, Thermo Fisher Scientific GmbH, Karlsruhe, Germany). The used material formulations are shown in Table 1. The compression molded plates (produced on a vacuum press Type P200PV, Dr. Collin GmbH, Ebersberg, Germany) were stored at 60 °C for a defined time and rheologically analyzed using a parallel plate rheometer (Physica MCR501, Anton Paar GmbH, Graz, Austria). The vacuum press was used to remove gases from the kneaded plates. 

#### Characterization of the Degree of Crosslinking of the Swelling Tests

Based on the results of the rheological investigations, the swelling tests were carried out on the samples which showed the best results with regard to the degree of crosslinking.

With the help of swelling experiments, crosslinking parameters such as gel content, swelling ratio, crosslinking degree, and molar mass M_c_ between the adjacent crosslinking points of crosslinked samples can be determined. In swelling tests, the samples are placed in a solvent in which they are soluble and after a certain time their weight was measured. Polyethylene is soluble in the solvent xylene at 80 °C. Non-crosslinked LDPE samples dissolve in this solvent, whereas crosslinked samples swell and largely retain their shape due to the crosslinked polymer chains [24]. The gel content is a measure of the proportion of insoluble material in the polymer. The higher the gel content, the higher the crosslinking density of the sample. The degree of swelling describes how strongly a sample swells. The lower the degree of swelling of a sample, the higher the crosslinking, and the lower the molecular weight between the crosslinks [25]. The crosslink density indicates the number of crosslinking points per volume unit.

The swelling tests were carried out according to ASTM D2765-01 [25]. Three samples of approx. 0.1 g each were taken at different locations of the crosslinked polymer plate. The samples were placed in a vessel with 12 mL of xylene for 24 h at a temperature of 80 °C. The samples were then weighed again and dried under vacuum at 80 °C for 24 h. The dried samples were then finally weighed. For each of the three samples, the crosslinking parameters were determined and the mean value calculated.

The gel content of the samples was determined by Equation (1) [26]:

(1) $$Gel\;content=\frac{w_{d}}{w_{i}}\cdot 100$$

with w_d_ as the dry weight after drying under vacuum at 80 °C and w_i_ as initial weight of the sample. 

The *swelling ratio* (Q) was determined by Equation (2) [27]:

(2) $$Swelling\;ratio=1+\left(\frac{w_{s}}{w_{d}}-1\;\cdot \;\frac{\rho_{p}}{\rho_{x}}\right)$$

with w_s_ as the weight of the swollen samples after storage in xylene, ρ_p_ as the density of the sample and ρ_x_ as the density of the solvent (0.879 g/cm³) [28]. The density of the sample was determined with a floating method.

Using the Flory-Rehner Equation (3), the *crosslinking density* (v_d_) of the individual polymers can be calculated [24]:

(3) $$v_{d}=-\frac{ln\left(1-Q^{-1}\right)+Q^{-1}+\chi_{1}Q^{-2}}{\phi_{1}\left(Q^{-\frac{1}{3}}-Q^{-\frac{1}{2}}\right)}$$

with Q as the swelling ratio. The flory interaction parameter (χ_1_) for LDPE extracted at 80 °C in xylene was taken from the literature reference with 0.49, while for the solvent volume (φ_1_) a value of 136 cm³/mol was taken [24].

Equation (4) was used to determine the molar mass between adjacent crosslinking sites (*M_c_*) [24]:

(4) $$M_{c}=\frac{\rho_{p}}{v_{d}}$$

with ρ_p_ as the density of the sample and v_d_ the crosslinking density of the sample. 

### 2.4. Foaming Experiments

The foaming experiments were performed on a grooved feed single screw extruder (Rosendahl Maschinen GmbH, Pischelsdorf, Austria) with a screw diameter of 45 mm and a length of 24D. The system was extended to a total length L of 32D with an 8D cylinder extension. A static mixer was also mounted before the die to improve the single-phase mixture. The extrusion die was a round die with a die diameter of 4 mm. The Teledyne Isco Syringe Pump 260D gas dosing station (Teledyne Isco Inc, Lincoln, Nebraska, USA) was used to inject the physical blowing agent. The blowing agent nitrogen was injected into the polymer melt at a rate of 0.05 wt%. The melt temperature was about 210 °C and all experiments were done with a screw speed of 10 rpm. The respective mixture was filled into the hopper of the single-screw extruder, and after having reached stationary conditions the injecting of the blowing agent was started. Only when stationary conditions were reached again were the samples collected carefully. The foamed samples were characterized in terms of density, mean cell size and cell density and compared with each other. The used material formulations are shown in Table 2.

#### Characterization of the Foamed Samples

The density measuring apparatus XS205 DualRange analytical balance from Mettler Toledo GmbH (Greifensee, Switzerland) was used to determine the foam density. This measuring apparatus is equipped with the density kit from Mettler Toledo GmbH. The density was determined by measuring the weight in two media of different density (air and water) (Archimedes principle).

The microscope Alicona InfiniteFocus (Alicona Imaging GmbH, Raaba/Graz, Austria) was used to determine the cell sizes. Three thin samples were cut from the foamed samples and examined under the microscope. Using the evaluation program, 20 bubbles were marked from each sample and the mean cross-sectional area was determined. The cell diameter was calculated on the assumption of an ideally round foam cell. The mean cell diameter of each individual sample was first determined using Equation (5). Then the mean cell diameter of all foamed samples was determined.

(5) $$D_{z,circle}=\;\sqrt{\frac{4A_{z}}{\pi }}$$

with D_z,circle_ as the diameter of the foam cells assuming a circular cross-section in µm and A_z_ as the area of the foam cell in µm².

The determination of the mean cell density (N_b_ in cells per cm³) was carried out according to Equation (6) [29]:

(6) $$N_{b}=\;\frac{1-\frac{\rho_{F}}{\rho_{m}}}{\frac{\pi }{6}\cdot D_{z,\;circle}^{3}}$$

with N_b_ as the average cell density in cells per cm³, ρ_F_ as the density of the foamed samples in kg/m³, ρ_m_ as the density of the non-foamed sample in kg/m³ and D_z, circle_ as the mean cell diameter in cm.

## 3. Results and Discussion 

### 3.1. Modification of Talcum

**Figure 2:**
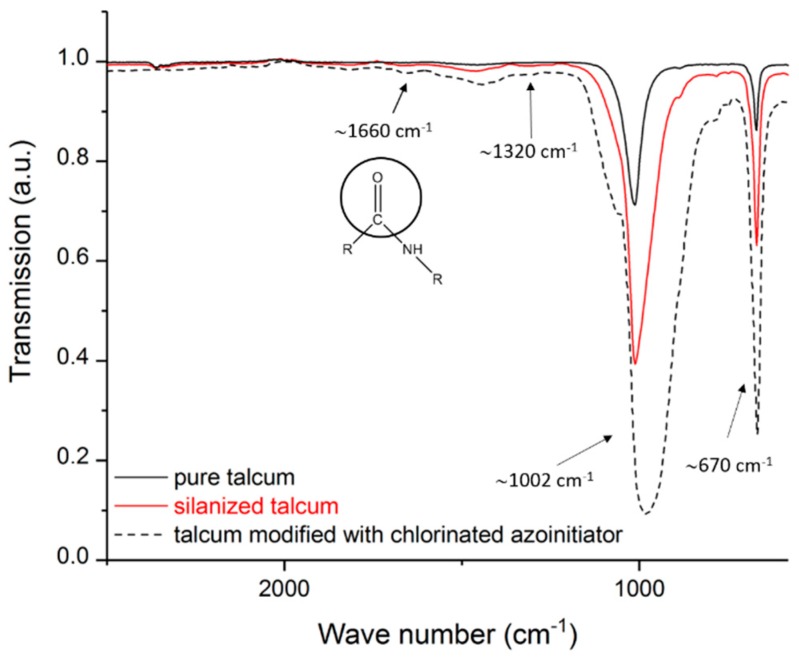
Infrared spectroscopy of pure, silanized and talcum modified with chlorinated azoinitiator.

**Figure 3:**
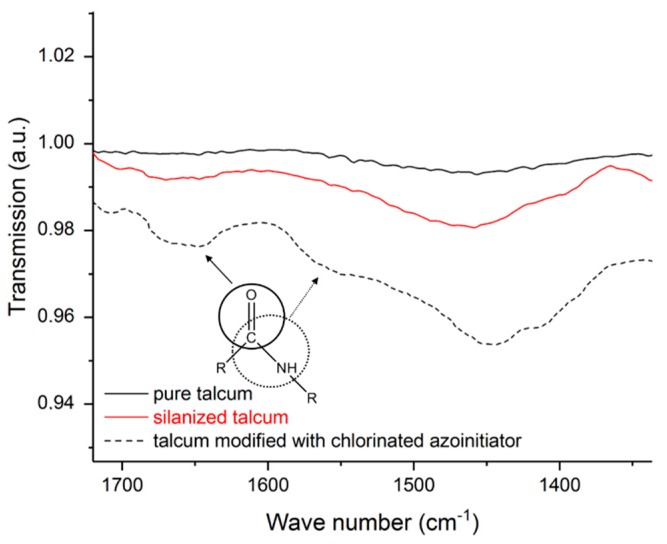
Selected ranges of infrared spectroscopy of pure, salinized, and talcum modified with chlorinated azoinitiator.

Before the modified talcum was used for supporting crosslinking and as nucleating agent the pure, the salinized and modified talcum was characterized. The size of the talcum particles and the associated smaller surface area relative to the mass led to a small amount of bound silane (or immobilized initiators) at the talcum surface. For this reason, some peaks are weakly pronounced. Figure 2 shows the spectra of the pure, the silanized, and the modified talcum with chlorinated azoinitiator. The IR spectrum of the pure talcum shows the characteristic stretching oscillations of the Si-O bands at 670 and 1002 cm^−1^ [30]. Due to the overlapping of the Si-O bands of the pure talcum with the Si-O compounds of the bound silane at the talcum surface of the silanized talcum, no unambiguous statement can be made here. However, the salinized talcum shows the typical stretching oscillation of the C-N compound at 1320 cm^−1^ [31]. This compound can be assigned to the used silane and is a proof for the coupling of the silane to the talcum surface. For the modified talcum, the oscillation of the carbonyl group of the carboxylic acid amides at 1660 cm^−1^ is visible [23,32]. These carbonyl groups arise because of the linkage between the thermal initiator and the NH_2_ groups of the aminosilanes. By enlarging certain regions of the IR spectra, the oscillation of the carbonyl group of the carboxylic acid amides at 1660 cm^−1^ (amide band I) is clearly visible (see Figure 3) [32]. In addition, a slight oscillation of the -CO-NH- group (amide band II) is visible at 1550 cm^−1^ [32]. The shift of the peak at ~1002 cm^−1^ is due to a change of the structure of the surface of the talcum.

**Figure 4:**
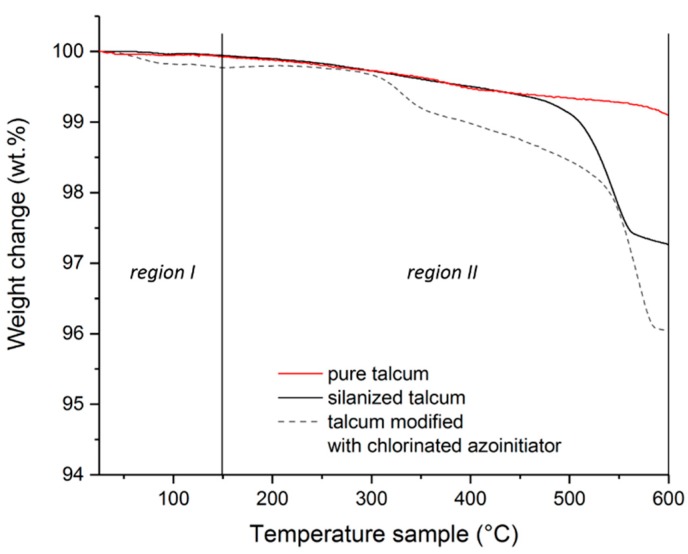
Thermogravimetric measurements of the pure, silanized and modified talcum.

Figure 4 shows the curves of the thermogravimetric measurements of the pure, the silanized and modified talcum. The curves were divided into 2 regions. Region I ranges from 25 to 150 °C and region II from 150 to 600 °C. The weight loss in region I is mostly due to adsorbed water or solvents still present. The loss in region II indicates the decomposition of organic groups. By comparing pure talcum and silanized talcum in region II, silanized talcum has a higher weight loss than the pure talcum. This mass change is due to the degradation of the organic groups of the silane [16,33,34,35]. The higher mass change of the modified talcum compared to the silanized talcum in region II is an indication that more organic groups have been decomposed. This is an indication of the immobilization of the initiator on the talcum surface. The mass change of the modified talcum in region I can be explained by physically bound volatile solvent residues.

### 3.2. Crosslinking Experiments

**Figure 5:**
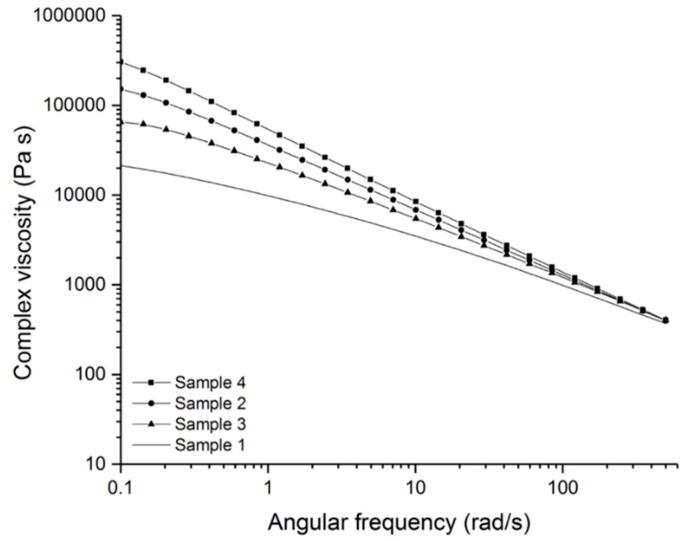
Complex viscosity of the crosslinked samples (sample 2–4) and the non-crosslinked sample (sample 1).

After the evidence of the linkage of the thermal initiator to the talcum surface, the polymer blends were kneaded and analyzed with a parallel plate rheometer using compression molded samples. The complex viscosity (η*), loss modulus (G’’) and storage modulus (G’) were determined for the crosslinked and non-crosslinked samples. Figure 5 shows the complex viscosity of the crosslinked samples (sample 2–4) and the non-crosslinked sample (sample 1). It can be clearly seen that all three material formulations (sample 2–4) exhibited a higher complex viscosity at a lower frequency range than the non-crosslinked sample after some days of aging. This is an indication of the crosslinking of the polymer. Due to the crosslinking of the polymer chains, the complex viscosity increased at low frequency ranges and showed nearly no Newtonian plateau [36]. When comparing the formulation with the pure talcum (sample 3) and the formulation without filler (sample 2), the sample without the talcum has a higher viscosity. The reason for this may be that the pristine filler hinders the attachment of the silanes to the chains or the covalent bond between the silanes and therefore leads to a lower crosslinking density. This causes a lower viscosity.

In Figure 5 it can be seen that the blend with the modified talcum (sample 4) shows the highest complex viscosity at lower angular frequencies. This result indicates that the formed radicals of the immobilized initiator support the crosslinking reaction and that the modified filler acts as an additional crosslinking agent. Furthermore, crosslinking can also take place between the polymer chains and the talcum and thus lead to a higher crosslinking density. The formed radical of the initiator bound to the talcum surface can form a bond with the silane and a siloxane compound (Si-O-Si) with the polymer chain. 

**Figure 6:**
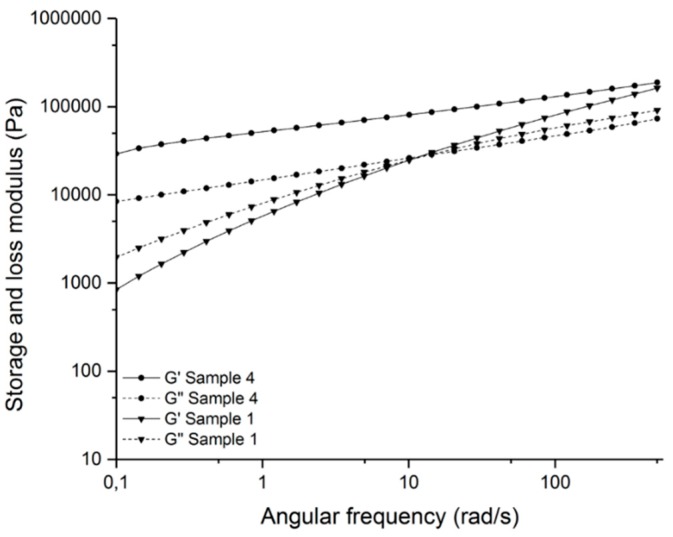
Storage and loss moduli (G’ and G’’) of the crosslinked sample (sample 4) and the non-crosslinked sample (sample 1).

By measuring the loss and storage moduli, the crosslinking network can also be determined (Figure 6). The crosslinked sample (sample 4) shows no cross over point of the loss and storage moduli, contrary to the non-crosslinked sample (sample 1). This indicates a crosslinking network [37].

**Figure 7:**
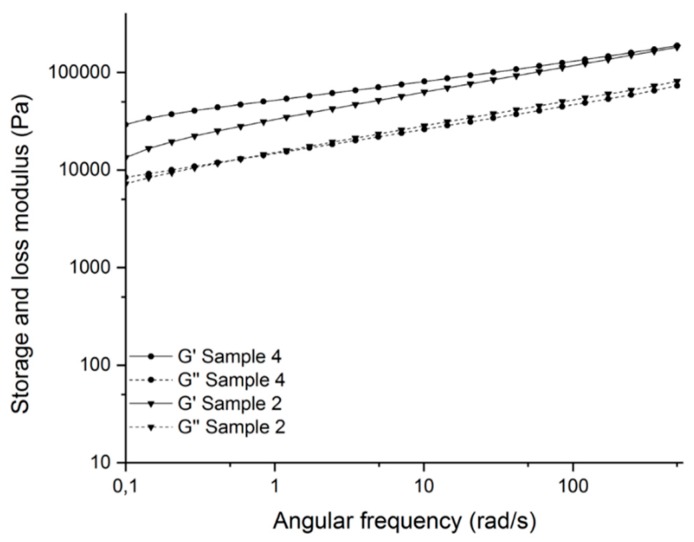
Storage and loss moduli (G’ and G’’) of the crosslinked samples with modified talcum (sample 4) and without talcum (sample 2).

Figure 7 shows the comparison of the storage and loss moduli of sample 4 with and sample 2 without the use of the modified talcum. At low angular frequencies, the sample with modified talcum shows a higher storage modulus than without. This effect can be explained by the restricted movement of the chains in the crosslinked network [38]. This indicates a better crosslinked network because of the additionally formed radicals of the initiator linked to the talcum surface. Details of the results are shown in Table 3.

Based on the results of the rheological investigations, the swelling tests were carried out on the samples, which showed the best results regarding highest zero viscosity and storage modulus (sample 4 and sample 2).

**Figure 8:**
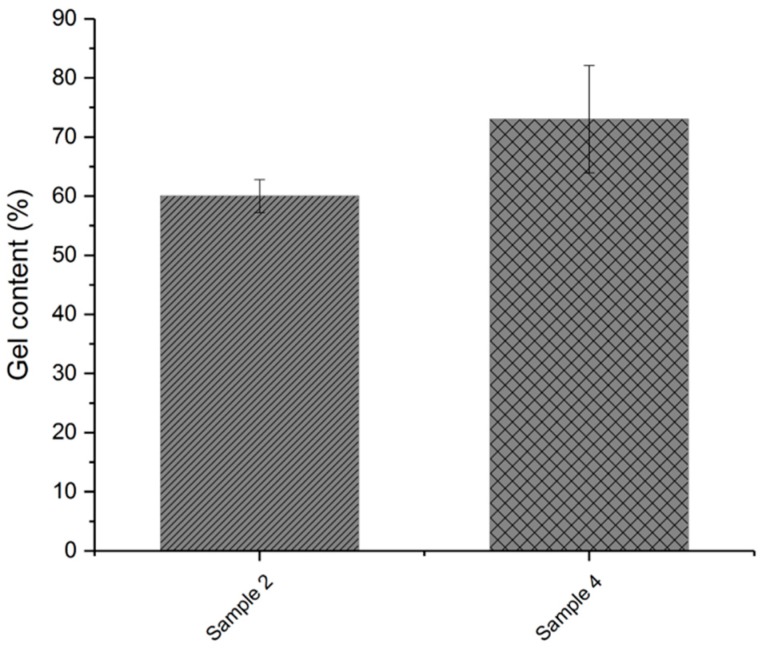
Gel content of crosslinked samples with modified talcum (sample 4) and without talcum (sample 2).

Figure 8 compares the results of the gel fraction of the formulation with talcum modified with chlorinated azoinitiator (sample 4) and the mixture without talcum (sample 2). As mentioned above, a higher gel content indicates a higher proportion of insoluble material in the polymer and is therefore an indication of a higher degree of crosslinking.

**Figure 9:**
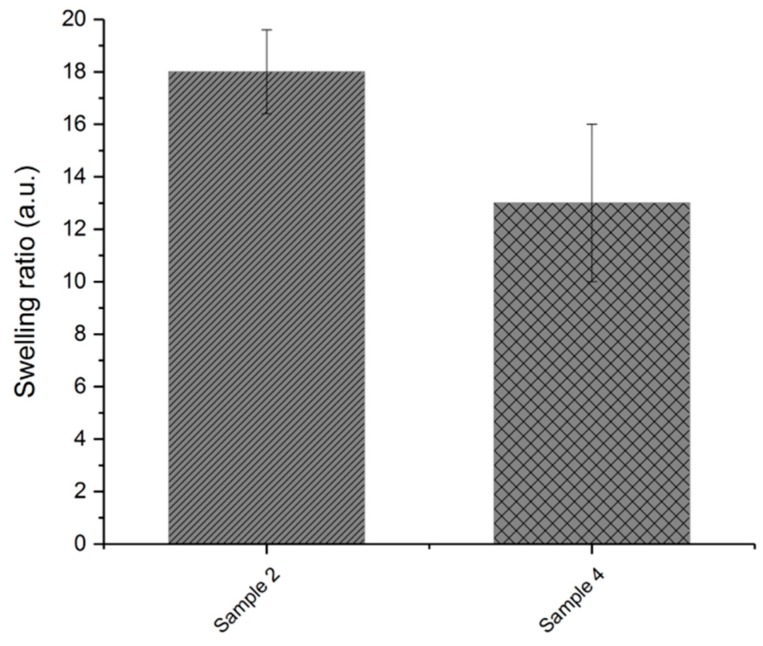
Swelling ratio of crosslinked samples with modified talcum (sample 4) and without talcum (sample 2).

**Figure 10:**
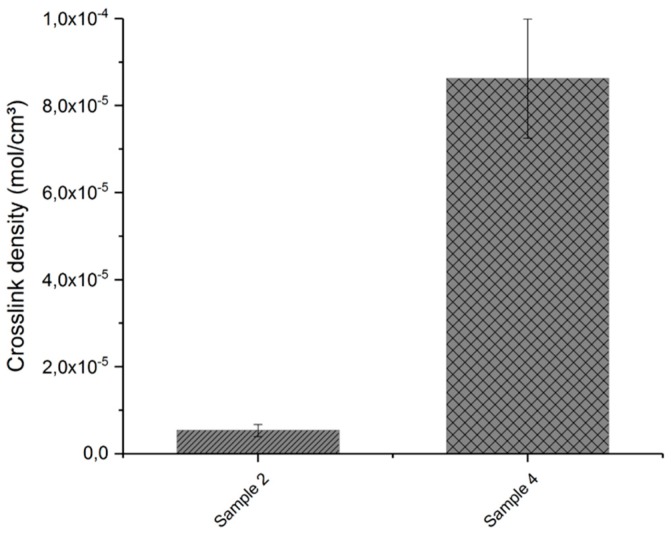
Crosslink density of crosslinked samples with modified talcum (sample 4) and without talcum (sample 2).

Figure 9 and Figure 10 show the swelling ratio and crosslink density of the crosslinked samples, respectively. From the results of the swelling ratio, it can be seen that the sample without talcum (sample 2) swells more than the sample with the modified talcum due to the solvent. This is an indication that the additional radicals of the initiator lead to a higher crosslinked polymer network. The crosslink density also indicates that the material formulation with the modified talcum has more crosslinking sites. 

In addition, the molar mass M_c_ of the crosslinked samples was calculated and is shown in Table 4. For the sample without talcum (sample 2), the molar mass between the adjacent crosslinks is higher and therefore the sample is less crosslinked. 

### 3.3. Foaming Experiments

**Figure 11:**
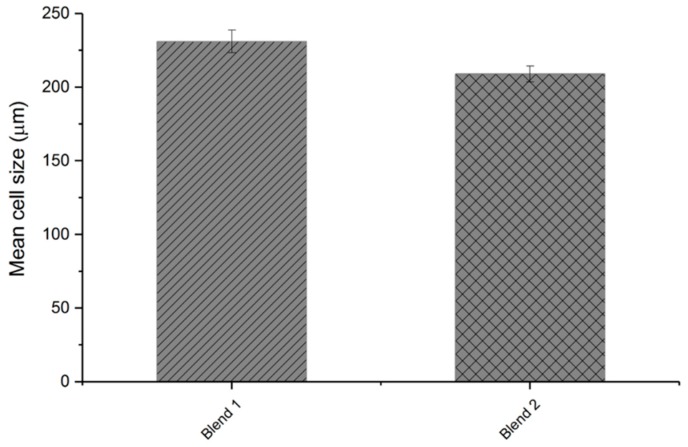
Mean cell size of foamed samples with pure talcum (blend 1) and modified talcum (blend 2).

In the next step, the influence of the modified talcum on the foam morphology was investigated. Figure 11 shows the mean cell size of the foamed samples. The blend with the modified talcum exhibits a better result regarding smallest cell size (blend 2).

**Figure 12:**
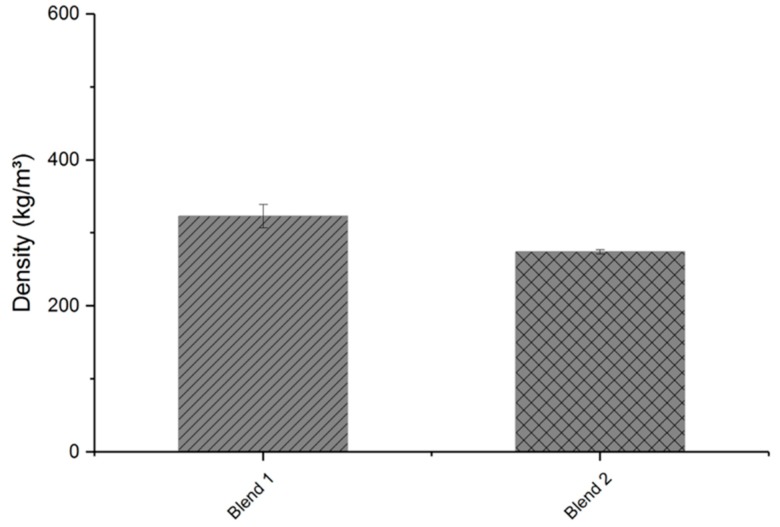
Density of foamed samples with pure talcum (blend 1) and modified talcum (blend 2).

The foam density of the foamed samples was also investigated. Figure 12 compares the results of the foamed samples with each other. Table 5 provides a comparison of the density results of the foamed samples and the unfoamed sample (blend 3). The highest density reduction is shown by the blend with modified talcum (blend 2). By using the modified talcum, not only smaller cells but also more cells were formed in comparison to the pristine nucleating agent. The results of small cell size and low foam density with blend 2 indicate that the decomposition of the initiator linked to the nucleating agent supports the foaming process. The additional gas formed near the nucleation sites may have led to a more uniform foam structure.

**Figure 13:**
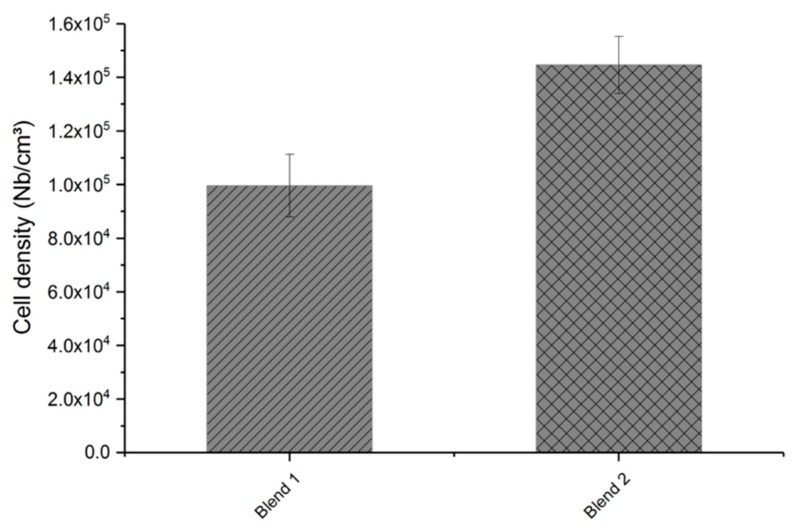
Cell density of foamed samples with pure talcum (blend 1) and modified talcum (blend 2).

**Figure 14:**
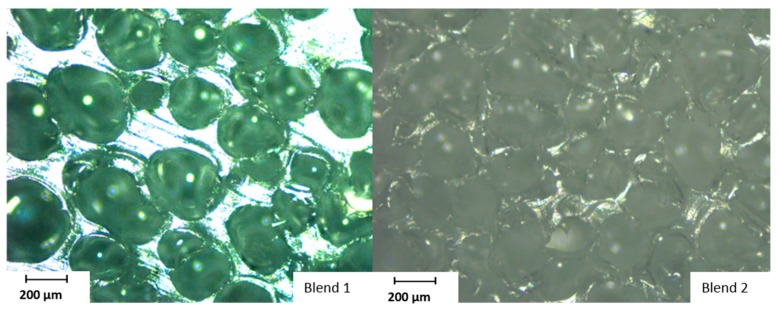
Foam structure of the blend with pure talcum (blend 1) and modified talcum (blend 2).

In the last step, the cell density of the foamed samples was calculated based on their cell diameter and foam density results. Figure 13 shows that an increase of cell density, and thus a higher number of cells per cm³, could be achieved with the modified talcum. Figure 14 shows the foam structure of the blend with the pure talcum (blend 1) and with the modified talcum (blend 2), measured with microscope Alicona InfiniteFocus.

## 4. Conclusions

In this study, the influence of talcum with modified surface for supporting crosslinking and as nucleating agent for foaming was investigated. The first step was the modification of talcum with the thermal initiator. With different measurements, it was possible to prove the linkage of the initiator to the talcum surface. After the modification of the nucleating agent, polymer blends with dicumyl peroxide, silane, and with the modified/pure and without talcum were produced and stored at 60 °C for a defined time. Afterwards, the samples were rheologically analyzed using a parallel plate rheometer. The results of the complex viscosity showed that the blend with the modified talcum showed the highest complex viscosity at lower angular frequencies. This indicates that the formed radicals of the immobilized initiator supported the crosslinking reaction and that the modified filler acted as an additional crosslinking agent. The loss and storage moduli of the crosslinked blends showed no cross over point, which is an evidence for crosslinked polymer chains. At low angular frequencies, the sample with modified talcum showed a higher storage modulus than without. This effect indicates a higher crosslinked network due to the additionally formed radicals of the initiator.

Additionally, the gel content, swelling ratio, crosslink density, and molar mass between adjacent crosslinking sites (M_c_) were investigated. These results indicate that the formed radicals of the thermal initiator linked to the talcum supported the crosslinking process and led to a better crosslinking network.

In the next step, the influence of the modified talcum on the foam morphology was investigated. The foamed samples with the modified talcum achieved a smaller average cell diameter and higher cell density compared to the use of the pure talcum. It seems that the additionally formed gas led to a more uniform foam structure and that the initiator linked to the talcum surface supports the foaming process.

By modifying the surface of talcum with an initiator, we found a possibility to support and improve the crosslinking process and foaming process of polyethylene.

## Figures and Tables

**Table 1:** Used material formulations for crosslinking experiments.

	Is Equal to 100 phr		Additives	
Sample	LDPE/EOC (Ratio)	1.6 wt%	Silane (phr)	Dicumyl Peroxide (phr)
Sample 1	80/20	-	-	-
Sample 2	80/20	-	4	0.5
Sample 3	80/20	pure talcum	4	0.5
Sample 4	80/20	talcum modified with chlorinated azoinitiator	4	0.5

**Table 2:** Used material formulation for foaming experiments.

	LDPE/EOC (Ratio)	Pure Talcum (wt%)	Modified Talcum (wt%)	N_2_ (wt%)
Blend 1	80/20	2	-	0.05
Blend 2	80/20	-	2	0.05
Blend 3	80/20	-	-	-

**Table 3:** Measured values of the zero viscosity (η0) and storage modulus (G‘) at an angular frequency of 0.1 rad/s.

Sample	η_0_ (kPa s)	G’ (kPa)
Sample 4	303.6	29.2
Sample 2	151.8	13.4
Sample 3	65.2	4.4
Sample 1	21.4	0.8

**Table 4:** Molar mass Mc of crosslinked samples.

Sample	Molar Mass M_c_ (g/mol)
Sample 2	2.1 × 10^5^
Sample 4	1.1 × 10^5^

**Table 5:** Density of the foamed samples (blend 1-2) and the unfoamed sample (blend 3).

Sample	Density (kg/m³)
Blend 3	902
Blend 1	323
Blend 2	274
